# Parental Catastrophizing and Goal Pursuit in the Context of Child Chronic Pain: A Daily Diary Study

**DOI:** 10.3389/fpsyg.2021.680546

**Published:** 2021-07-01

**Authors:** Line Caes, Cynthia van Gampelaere, Eline Van Hoecke, Myriam Van Winckel, Kristien Kamoen, Liesbet Goubert

**Affiliations:** ^1^Division of Psychology, Faculty of Natural Sciences, University of Stirling, Stirling, United Kingdom; ^2^Department of Experimental, Clinical and Health Psychology, Faculty of Psychology and Educational Sciences, Ghent University, Ghent, Belgium; ^3^Pediatric Psychology, Department of Pediatrics, Ghent University Hospital, Ghent, Belgium; ^4^Department of Internal Medicine and Pediatric, Faculty of Medicine and Health Sciences, Ghent University Hospital, Ghent, Belgium; ^5^AZ Maria Middelares, Ghent, Belgium

**Keywords:** chronic pain, parents, diary, catastrophizing, goals, distress

## Abstract

**Background:** Despite daily variability in children's chronic pain experiences, little is known about how parents' emotions and goals toward their child's pain are influenced by these daily changes. This diary study examined how daily child pain intensity (as perceived by parents) moderates the associations between parental catastrophic thoughts about child pain on the one hand, and daily parental distress and parents' goals with regard to their child's pain (pain control vs. activity engagement) on the other hand.

**Method:** Participants were 25 parents of 20 different children *(N* = 18; 90% girls). Children, aged 8–14 years (*M* = 9.5, *SD* = 2.09), experienced either chronic headache or functional abdominal pain with an average pain duration of 22.5 months (*SD* = 24.5 months). Daily parental responses (i.e., perceived child pain intensity, distress and goal endorsement) were collected through a 3-week daily diary (resulting in 413 valid diary reports). Parents completed the Pain Catastrophizing Scale for Parents prior to starting the diary (PCS-P general) and a daily measure (PCS-P daily) included in the diary. To account for the interdependence of the data, the data were analyzed using multilevel modeling.

**Results:** Perceived daily child pain intensity moderated the impact of parental general and daily catastrophic thoughts on parents' daily distress. Only for parents experiencing low general catastrophic thoughts an increase in distress was observed on days when they perceived their child's pain intensity as high. For all parents, high levels of perceived child pain intensity were related to more distress on days where parents reported high levels of catastrophic thinking (i.e., PCS-P daily). Perceived daily child pain intensity also moderated the impact of parental general catastrophic thinking on parents' daily endorsement of goals. Parents with high levels of general catastrophic thinking reported a lower focus on child pain control on days when child pain intensity was perceived to be low. Parents with low general catastrophic thinking reported lower endorsement of the activity engagement goal on days where the child's pain intensity was perceived to be low.

**Conclusion:** These findings highlight the complexity of daily fluctuations in parental distress and goals regarding their child's pain. Clinical implications and future directions are critically assessed.

## Introduction

Chronic pain in children is a common and serious health- and developmental problem that has a major impact on the child's daily living (Palermo, 2000; Oddson et al., 2006). Prevalence of chronic pain in children ranges between 11 and 38%, increases with age, and is higher in girls as opposed to boys (Perquin et al., 2000; King et al., 2011). The most frequently examined types of chronic pain in children are abdominal pain, headache, back pain, and musculoskeletal pain (King et al., 2011). Children who report a high frequency of pain and high current pain generally report a lower quality of life (Oddson et al., 2006). Furthermore, children experiencing chronic pain have a higher risk for developing psychiatric disorders, especially anxiety disorders or depression, compared to children without chronic pain complaints (Palermo, 2000; Machnes-Maayan et al., 2014). However, many children function well despite the presence of chronic pain. Whether and how much pain-related disability a child with chronic pain experiences, depends, among other things, on several child characteristics. For example, children with higher levels of anxiety sensitivity and fear of pain report more pain-related disability (Martin et al., 2007). However, to fully understand children's pain-related disability it is crucial to also take into account the role of how parents respond to and cope with their child's pain experiences (Goubert and Simons, 2013).

Childhood chronic pain can be considered a substantial stressor for parents for which they need to find appropriate coping approaches to avoid or minimize pain-related disability. According to the Interpersonal Fear Avoidance model (IFAM; Goubert and Simons, 2013), a pain-specific cognitive process, important to understand how parents cope with and respond to their child's pain, is catastrophic thinking (Goubert et al., 2006). Catastrophic thinking is the tendency to focus upon the threat value of the pain stimuli, to exaggerate the threat value and to negatively evaluate one's own (or one's child's) abilities to handle the pain (Sullivan et al., 2001). Parental catastrophic thinking is thus an exaggerated negative mental set or response that emerges during painful experiences of one's child and might have negative consequences. Catastrophic thinking can be assessed as either a trait or state characteristic, both of which will be examined within this study. We will use the term “general” to refer to trait levels of catastrophizing and “daily” for state levels of catastrophizing. There is, indeed, an abundance of evidence supporting the assumptions of the IFAM by showing how parental catastrophic thinking is related to heightened feelings of distress and maladaptive coping responses, such as pain-attending/protective behaviors, which in turn are related to more pain and disability in children (Goubert and Simons, 2013).

To enhance understanding of parental coping approaches and their impact on child functioning, comprehension of parents' underlying goals is needed. Based on the tenets of motivational theories on goal-directed behavior (Riediger and Freund, 2004), it is likely to assume that parents have multiple goals for their children, such as pain relief, attending school, socializing with friends, and engaging in leisure activities (e.g., sports, arts; Rasmussen et al., 2006). Not all goals might be compatible with each other, in which case parents need to prioritize some goals over other (Riediger and Freund, 2004). In line with the above-described evidence, parents with high levels of catastrophic thinking might be more protective toward their child in pain because they prioritize the goal to relieve their child's pain over the other goals they have for their child (Caes et al., 2012). However, little is known as to why this is the case. To gain a better understanding of parental coping mechanisms to deal with their child's pain, more research is needed to explore parental goals when faced with child (chronic) pain, and how the endorsement of different goals varies depending on daily child pain characteristics. On a daily basis, parents may want to pursue the goal to control or relieve their child's pain as well as the goal to encourage their child toward engagement in daily activities (such as attendance at school or leisure activities) in the presence of pain. While for some occasions, both goals could be achieved through engagement in the same coping approach (e.g., promoting distraction), in other circumstances this might not be possible leading parents to perceive these two goals as potentially incompatible. Hence, a careful balance in pursuing these two goals may have important clinical consequences in terms of adequate child functioning and development.

As any goal, the goal of controlling pain can be attained by different parental responses, such as comforting or distracting their child (Carver and Scheier, 2001; Riediger and Freund, 2004; Rasmussen et al., 2006). The adaptive or maladaptive impact of different parental behaviors upon child functioning might depend on the extent to which behavior is primarily and inflexibly driven by the parental goal for pain control at the expense of other important aspects/goals in their child's life. Specifically, although the use of coping strategies, such as distraction, could be motivated by the goal of controlling child pain, engaging in distraction may also reflect parental attention for other aspects of child functioning despite the pain. This could explain the positive influence of this coping strategy on child functioning (Gonzalez et al., 1993; Sweet and McGrath, 1998; Blount et al., 2008; MacLaren Chorney et al., 2009). In contrast, parental protective responses, such as allowing the child to stay home from school, may reflect a strong priority of parents to reduce pain even if this negatively impacts their child's daily functioning substantially. Further research is needed to investigate how parents flexibly attune between child pain needs (i.e., pain control) and non-pain needs and how this translates into behavior.

Given the fluctuating nature on a daily basis of chronic pain and the mutual influence between child pain characteristics and parental responses proposed in the IFAM (Goubert and Simons, 2013), it is plausible to assume that the way parents attune to their child's needs is likely to fluctuate and depend on daily pain characteristics of the child. Indeed, preliminary evidence, using a vignette methodology (Caes et al., 2012), revealed that higher levels of child pain intensity are related to parents attaching more priority to pain control goals. Nevertheless, research on the role of catastrophic thinking in understanding how parents cope with their child's pain is limited on focusing mostly on general tendencies, while little is known about the daily fluctuations in parental catastrophic thinking and its subsequent impact on parental distress and behavioral tendencies/underlying goals. The usage of diary methodologies is lacking in this context but could offer a unique insight into and capture these daily fluctuations in childhood pain experiences and how this impacts parental coping mechanisms on a daily basis.

**Figure 1:**
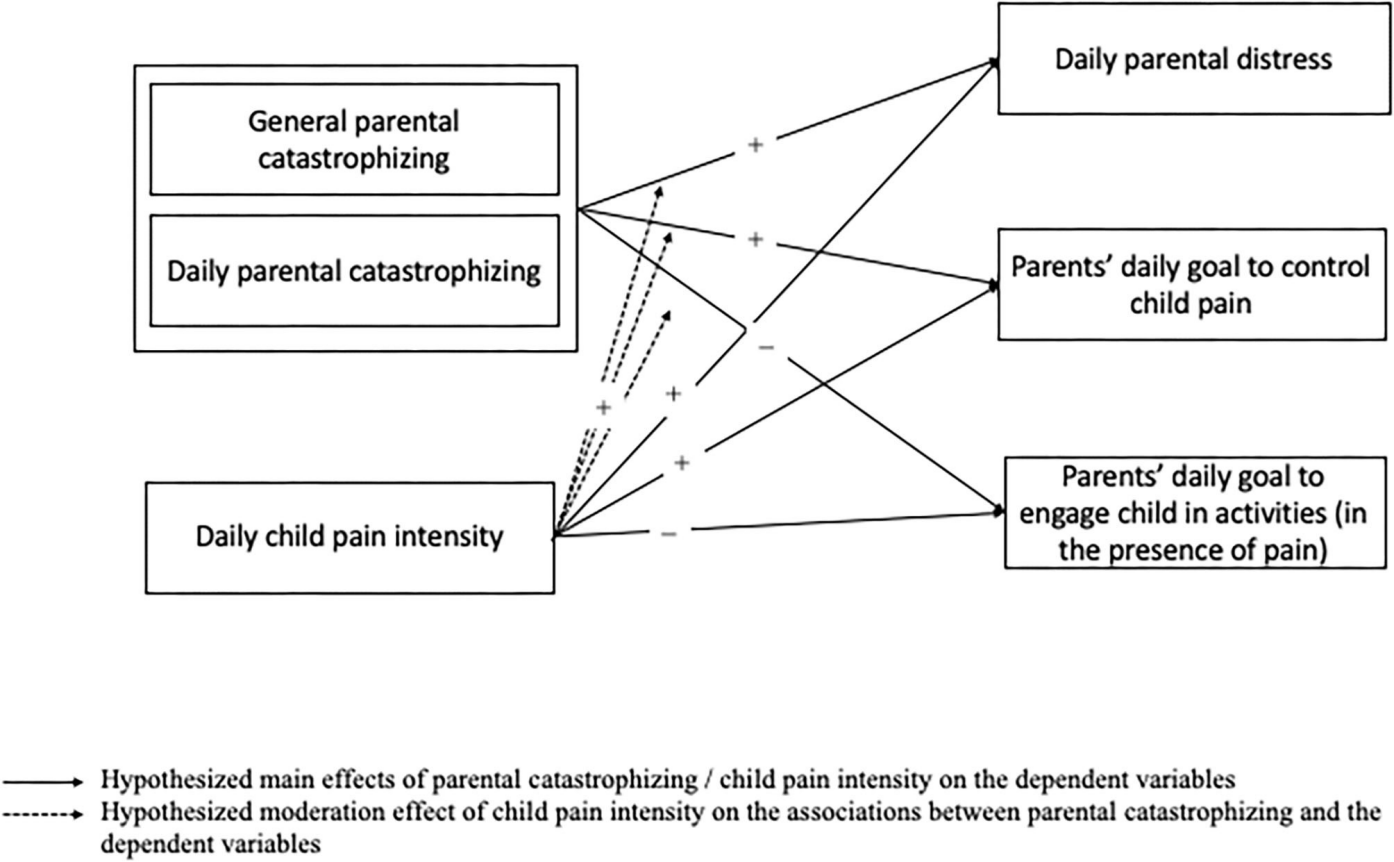
Hypothesized main and moderation effects.

Consequently, the primary aim of the current diary study, in children (8–14 years) with chronic headache or chronic functional abdominal pain, was to examine the associations between parental catastrophizing about the pain of their child, both in general and on a daily level, on the one hand, and daily parental distress and daily parental goals of child pain control vs. child activity engagement on the other hand. We expected that parents with high levels of catastrophic thoughts about their child's pain, compared to parents low on catastrophic thinking, would (1) experience more daily distress, (2) endorse the goal to relieve the pain of their child to a higher extent on a daily basis, but the goal to engage their child in activity engagement (in the presence of pain) less. While no difference in the direction of the associations was expected for general vs. daily parental catastrophic thinking, we expected that the hypothesized associations would be stronger for daily levels of catastrophizing compared to general levels (Durand et al., 2017; see Figure 1). As a second aim, the role of daily child pain intensity, as reported by the parent, was examined (further referred to as “perceived daily child pain intensity”). We expected perceived daily child pain intensity, to be (1) positively associated with daily parental distress and the daily parental goal to control their child's pain, and (2) negatively associated with the daily parental goal to engage their child in activity engagement. Furthermore, we hypothesized that higher perceived child pain intensity would strengthen the associations between (general and daily) parental catastrophizing on the one hand, and parental distress and the extent to which parents endorse child pain control or activity engagement goals on the other hand (see Figure 1).

## Materials and Methods

The study is part of the G-GiCPP project (Ghent—Goals in Chronic Pediatric Pain—Project) that consists of two parts; a cross-sectional questionnaire part, followed by an optional 3-week diary part, completed by both children with chronic pain and their parents. The current manuscript focuses on reporting the findings from the diary as completed by the parents and on parental catastrophizing as measured during the questionnaire study prior to starting the diary. The study was approved by the Ethics Committees of the Faculty of Psychological and Educational Sciences of Ghent University, University Hospital Ghent, AZ Maria Middelares Ghent, and General Hospital Nikolaas, Belgium.

### Participants

Families with children with chronic headaches or chronic functional abdominal pain were recruited through four Flemish hospitals (University hospital Ghent, Maria Middelares Ghent, Jan Palfijn Gent, general hospital Nikolaas). To be eligible for study participation, children had to be aged between 4 and 16 years, children had to be diagnosed by their physician with chronic headache or chronic functional abdominal pain, and both parent and child had to be Dutch-speaking. Children with a developmental disorder, mental delay or migraine were excluded. Families who met the inclusion criteria were informed about the study by their physician. Eighty-two families agreed to be contacted for study participation, of which 48 families (58%; with data from 62 parents) participated in the first part of the study (i.e., the questionnaire part). The main reason for non-participation was parent-reported lack of time. Of the 48 families (covering 62 parents) that completed the questionnaires, 24 families (50%; with data from 32 parents, 52%), also completed the diary part. Due to methodological reasons (i.e., < completed <50% or 11 days of the diary entries) 7 parents were excluded from the analyses. The final sample consisted of 25 parents (5 mother-father dyads, 14 mothers only, 1 father only) of 20 different children. Demographics of the final sample of 25 parents, across 20 children, are presented in Table 1.

**Table 1:** Demographic characteristics of the final sample of 25 parents, across 20 children.

		***N* (%)**	**Mean**	**Range**	***SD***
**Child (*****N*** **=** **20)**					
Age (years)			9.7	8–14	1.49
Pain duration (months)			22.53	2–96	24.36
Type of pain	Chronic headache	5 (25.0%)			
	Chronic functional abdominal pain	14 (70.0%)			
	Unknown	1 (5.0%)			
Medication	Takes medication	11 (55.0%)			
	Pain medication	5 (25.0%)			
	No medication	9 (45.0%)			
Nationality	Belgian	20 (100%)	
Gender	Girls	18 (90.0%)	
	Boys	2 (10.0%)	
Marital status of parents	Married/cohabiting	18 (90.0%)	
	Divorced	1 (5.0%)	
	Blended family	1 (5.0%)	
**Parent (*****N*** **=** **25)**					
Age			41.03	35–48	3.56
Gender	Mother	20 (76.9%)	
	Father	6 (23.1%)	
Education	Highly educated (>18 years)	20 (76.9%)	
	High school	4 (15.4%)	
	Middle school	2 (7.7%)	

### Data Collection Procedure

All families who were interested in participation and gave written consent to transfer their contact details to the researchers received a phone call with further detailed information about the study. If after receiving all necessary information parents were interested to participate, a home visit was scheduled. During the home visit, the study information was repeated, and parents provided informed consent for themselves and for the child. Children older than 12 years provided, in addition, written informed consent for themselves. In a first phase all parents and children (≥9 years) completed questionnaires under supervision of a research assistant and the procedure of the diary study was explained. Following the home-visit, parents were sent an email containing a secured weblink to the online diary (LimeSurvey software). Parents were asked to complete the diary every evening for 21 consecutive days. Diaries were completed during school weeks and started shortly after the home visit. All families received 30 EUR as a compensation for participation-related costs.

### Questionnaire Measures

*General parental catastrophizing about their child's pain* was assessed by the Dutch Pain Catastrophizing Scale for Parents (general PCS-P; Goubert et al., 2006), an adaptation of the adult Pain Catastrophizing Scale (PCS; Sullivan et al., 1995). The PCS-P encompasses 13 items, rated on a 5-point Likert Scale (0 = not at all, 4 = extremely). Parents were asked to rate how frequently they experience different thoughts and feelings when their child is in pain (e.g., “When my child is in pain, I become afraid that the pain will get worse”). The PCS-P contains three subscales, assessing *rumination* (ruminate about the pain), *magnification* (the thought that something serious will happen due to the pain), and *helplessness* (the feeling that you cannot stand it anymore because of the pain), and yields a total score that ranges between 0 and 52, with higher scores indicating more catastrophic thoughts. The PCS-P demonstrated adequate internal consistency in the current study (α = 0.90).

### Diary Measures

Below is an overview of the constructs assessed by the diary. All diary items were rated on a 7-point Likert-scale (for example, 0 = not at all, 6 = a lot). For constructs assessed by multiple items, the mean of the respective items was calculated, resulting in a total score ranging from 0 to 6. Level-specific reliabilities were estimated based upon a multilevel confirmatory factor analysis framework. Within-parent, between-parent, and between-couple alphas are reported in Table 2 (Geldhof et al., 2014).

**Table 2:** Level-specific reliabilities of the diary variables.

**Construct**	**Within parent α**	**Between parent α**	**Between couple α**
Parental distress when confronted with the child's pain	0.77	0.94	0.85
Parental catastrophic thinking about their child's pain	0.82	0.83	0.88

*Reliabilities were estimated by a multilevel confirmatory factor analysis framework (Geldhof et al., 2014)*.

*Perceived daily child pain intensity* was reported by the parents and measured by one item: “How much pain did your child experience today, on average, according to you?”

*Daily parental distress when confronted with their child's pain* was assessed by means of seven items, based upon the theory of Batson et al. (1987). Parents were asked to report to what extent they experienced different feelings that day, related to the pain of their child (i.e., “Indicate to what extent you experienced the emotions listed below today in response to your child's headache or abdominal pain: worried, anxious, upset, sad”).

*Daily parental catastrophic thoughts about their child's pain* was measured by means of the PCS-P daily (Durand et al., 2017), which includes a selection of 3 items from the PCS-P (Goubert et al., 2006) adapted for use in a daily context. For each subscale of the PCS-P, the PCS-P daily contains one item (i.e., Rumination: “Today, to what extent did you kept thinking about how much pain your child experienced?”; Magnification: “Today, to what extent did you think that, because of the pain, something serious might happen to your child?”; Helplessness: “Today, to what extent did you think, because of the pain of your child, you would not be able to stand it anymore?”).

*Daily parental goals of child pain control and child activity engagement* were assessed by asking parents to report on the degree to which they focused on either relieving their child's pain “To what extent were you focused on relieving your child from his/her pain today?”) or encouraging their child to engage in activities (i.e., “To what extent did you encourage your child to engage in his/her daily activities today, even if he/she could experience pain by doing so?”). The items were formulated based on the items used in the vignette study by Caes et al. (2012), which used adjusted items of the Chronic Pain Acceptance Questionnaire (CPAQ-8; Fish et al., 2010). We know of no other existing questionnaire measuring parental goal engagement in the context of pain.

Parents completed in total 485 end-of-day diary observations. In case of multiple records on the same day, the first completions were deleted (*N* = 4). Diaries completed after 10 AM the next day or before 4 PM the same day were deleted (*N* = 16) (Nezlek, 2012). The records of 7 participants (for a total of 65 records) were excluded from the analyses due to completing less than half of the requested 21 entries (< 11 valid records). Finally, 413 records were included in the analyses, representing 85% of the available records and including reports across 25 parents. On average, parents completed 16 diaries (range 12–21).

### Analyses

The data of the present study are hierarchically nested within 3 levels of assessment: perceived daily pain intensity, parental daily catastrophic thoughts, daily distress, and daily pain control and activity engagement goal (Level 1) are nested within the participating parents (Level 2), which are nested within a particular family (Level 3). To account for this interdependence and hierarchical nesting of the data, the data were analyzed by means of multilevel modeling (HLM version 6.01, Raudenbush et al., 2004). Multilevel modeling is the preferred method for this data structure as it allows more precise parameter estimates compared with more traditional statistical methods (e.g., repeated-measures analyses of variance; Kenny et al., 2006, Nezlek, 2001), and can deal appropriately with missing data (Hox, 2010). Importantly, multilevel modeling allows for a mother and father of the same child to be identified as different participants while accounting for the dependency in their data due to being parents of the same child (Kenny et al., 2006).

For each of the three dependent variables (i.e., parental distress, pain control goal pursuit and activity engagement goal pursuit) the same set of analyses was performed to test the hypotheses. In a first step, a baseline model, without any predictors was run to calculate the level of variance in the dependent variables accounted for by the variables between parents (Level 2) and within parents (Level 1). In the second step, we added the Level 3 control variables child age, child sex and pain duration. These demographic variables were included in the models as evidence in the literature highlights that pain experiences, and parental responses vary depending on the child's age, sex and pain duration (Cohen et al., 2010; Palermo et al., 2014; Boerner et al., 2015). The categorical variable child sex was dummy coded (0 = male; 1 = female) and added to the model uncentered. The continuous variables child age and pain duration were standardized before adding to the model. In the third step, the impact of Level 1 variables was assessed by adding the standardized, group-mean centered scores for PCS-P daily and perceived daily child pain intensity. In the fourth step, the influence of parental general catastrophic thinking was evaluated by adding the standardized, grand-mean centered Level 2 variable PCS-P general. In the last and fifth step, the slopes for Level 1 variables were set to random on Level 2 and if the random error term was significant, two interaction terms were added: the cross-level interaction between perceived child pain intensity and PCS-P general and the Level 1 interaction between perceived child pain intensity and PCS-P daily. If the random error term was not significant (*p* < 0.05; Nezlek, 2001), the slopes were fixed. The slopes for all Level 1 and 2 variables were fixed on the third level because dyads do not have enough lower-level units to allow for the slopes to vary (Kenny et al., 2006). Full maximum likelihood estimation was applied for each step in this build-up strategy. The effect size *r* was calculated for all significant effect, with an *r* value of 0.10 reflecting a small effect, an *r* value of 0.30 reflecting a medium effect and an *r* value of 0.50 reflecting a large effect (Kenny et al., 2006).

## Results

### The Impact on Daily Parental Distress

The intercept model indicated that 11.22% of the variance in parental distress was accounted for by variables on the third level (between families or child characteristics), 62.78% by variables on the second level (parent characteristics), and 38.32% by variables on the first level (within parents or daily characteristics). The model exploring the main effects of the variables across all levels (steps 2–4) revealed a significant positive effect of daily parental catastrophic thinking (PCS-P daily) [γ_200_ = 0.40; *t*_(352)_ = 11.36; *p* < 0.001, *r* = 0.41] and perceived daily child pain intensity [γ_100_ = 0.22; *t*_(352)_ = 5.76; *p* < 0.001, *r* = 0.23]. No main effect of general parental catastrophic thinking (PCS-P general) was found. The random error terms for perceived daily child pain intensity and PCS-P daily were significant, so the interaction terms (perceived daily pain intensity^*^PCS-P daily and perceived daily pain intensity^*^PCS-P general) were added in step 5. Both interaction terms were significant.

**Figure 2:**
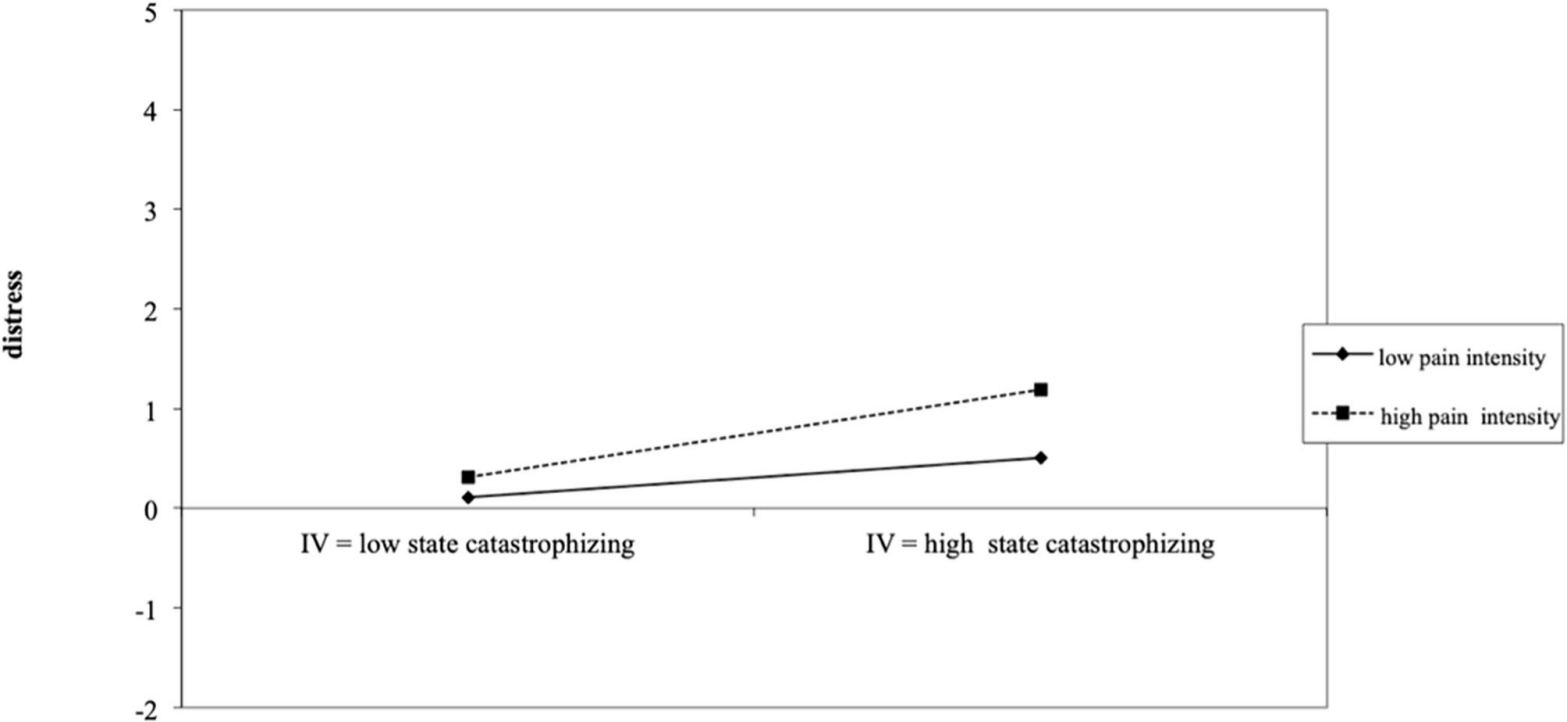
Depiction of how the significant interaction between daily parental catastrophizing and perceived daily child pain intensity influences parental daily distress experiences.

A significant positive interaction was found between daily levels of parental catastrophizing and perceived daily child pain intensity [PCS-P daily; γ_300_ = 0.11; *t*_(350)_ = 6.47; *p* < 0.001, *r* = 0.26]. This interaction reveals that on days that parents catastrophize a lot about their child's pain, their distress is strongly influenced by the daily level of perceived child pain intensity with higher levels of perceived child pain intensity related to more parental distress. On days that parents experience low levels of catastrophic thoughts; parental distress remains lower compared to days on which parents catastrophize a lot and the perceived level of the child's pain intensity does not add much to explaining this distress level (See Figure 2).

**Figure 3:**
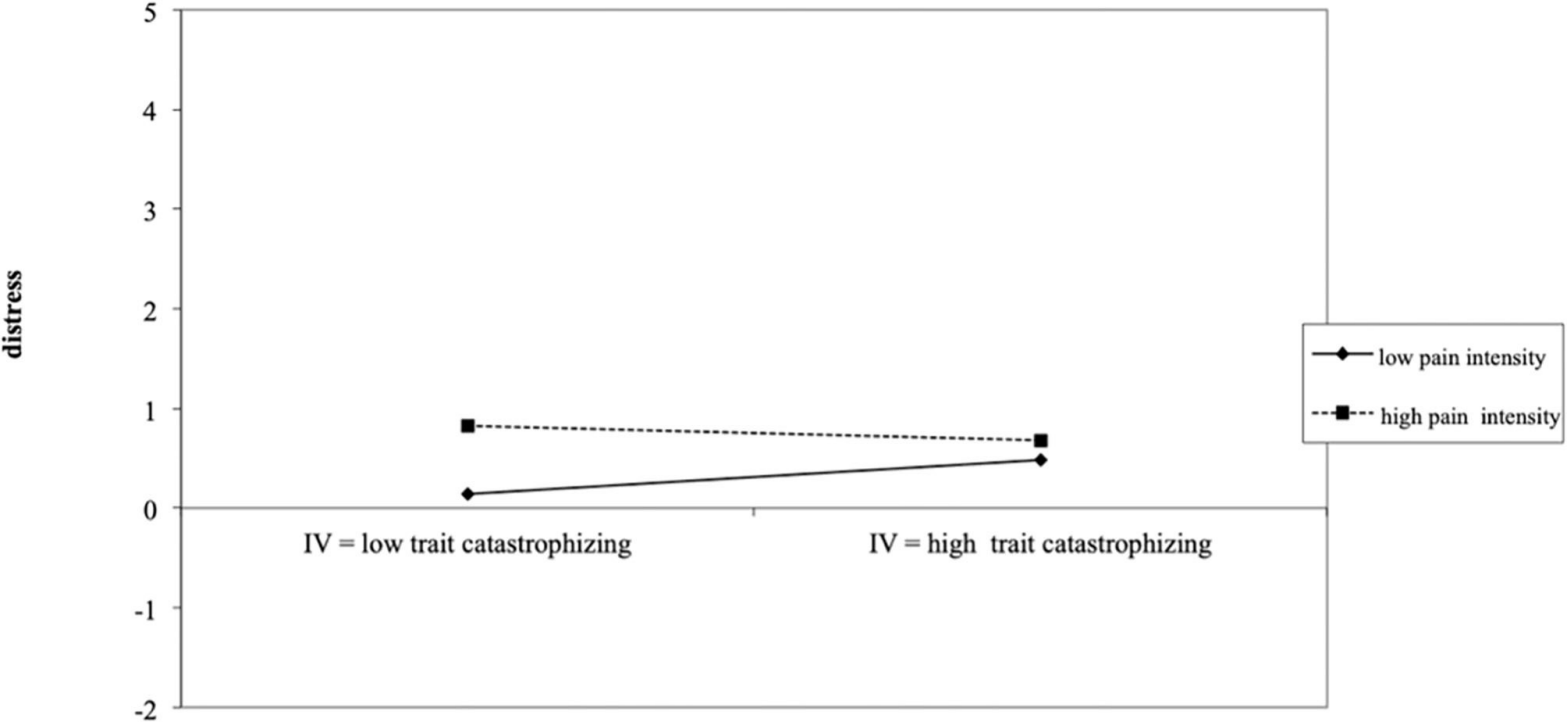
Depiction of how the significant interaction between daily parental catastrophizing and perceived daily child pain intensity influences parental daily distress experiences.

In contrast, a significant negative interaction between general levels of parental catastrophic thoughts and perceived daily child pain intensity [PCS-P general; γ_110_ = −0.12; *t*_(350)_ = −4.26; *p* < 0.001, *r* = 0.18] was found. This finding indicates that for parents with low general levels of catastrophic thoughts about their child's pain, increasing levels of perceived daily child pain intensity were related with increased levels of daily parental distress. However, for parents with high general levels of catastrophic thinking, the perceived level of daily child pain intensity did not add much explanation to the experienced level of daily parental distress (see Figure 3). Results for the final hierarchical linear model assessing the impact of parental catastrophic thinking and perceived child pain intensity on parental distress are presented in Table 3.

**Table 3:** Final hierarchical linear models of daily parental distress, endorsement of pain control goal and endorsement of activity engagement goal.

	**Parental daily distress**			**Parental endorsement of pain control goal**			**Parental endorsement of activity engagement goal**		
**Variable**	**β coeff**.	**SE**	**T**	**β coeff**.	**SE**	**T**	**β coeff**.	**SE**	**T**
Intercept (γ000)	0.16	0.25	0.64	2.46	0.36	6.85^***^	4.48	0.94	4.75^***^
Child sex (γ001)	0.40	0.27	1.45	−1.09	0.39	−2.78^*^	−2.17	1.01	−2.14^*^
Child age (γ002)	0.21	0.11	1.90^#^	0.11	0.16	0.66	0.12	0.42	0.30
Pain duration (γ003)	0.02	0.07	0.33	0.11	0.11	1.07	0.11	0.28	0.39
PCS-P general (γ010)	0.05	0.09	0.56	−0.08	0.12	−0.68	0.13	0.30	0.42
PCS-P general^*^ Perceived pain intensity (γ110)	−0.12	0.03	−4.26^***^	0.15	0.06	2.39^*^	−0.30	0.08	−3.59^***^
Perceived pain intensity (γ100)	0.22	0.04	6.08^***^	0.94	0.08	11.75^***^	−0.42	0.11	3.87^***^
PCS-P daily (γ200)	0.32	0.04	8.18^***^	0.41	0.09	4.72^***^	0.32	0.12	2.74^**^
Perceived pain intensity^*^PCS-P daily (γ300)	0.12	0.02	6.47^***^	−0.02	0.04	−0.44	0.09	0.05	1.61

# *p = 0.08,*

* *p < 0.05,*

** *p < 0.01,*

*** *p < 0.001*.

### The Impact on Parents' Daily Endorsement of the Pain Control Goal

The intercept model indicated that 25.57% of the variance in the pain control goal was accounted for by variables on the third level (between families or child characteristics), 73.05% by variables on the second level (parent characteristics), and 1.38% by variables on the first level (within parents or daily characteristics). The model exploring the main effects of the variables across all levels (steps 2–4) revealed a significant positive effect of daily parental catastrophic thinking (PCS-P daily) [γ_200_ = 0.43; *t*_(352)_ = 5.87; *p* < 0.001, *r* = 0.30] and perceived daily child pain intensity [γ_100_ = 0.96; *t*_(352)_ = 12.07; *p* < 0.001, *r* = 0.54]. No main effect of general parental catastrophic thinking (PCS-P general) was found. The random error terms for perceived daily child pain intensity and PCS-P daily were significant, so the interaction terms (perceived daily pain intensity^*^PCS-P daily and perceived daily pain intensity^*^PCS-P general) were added in step 5.

**Figure 4:**
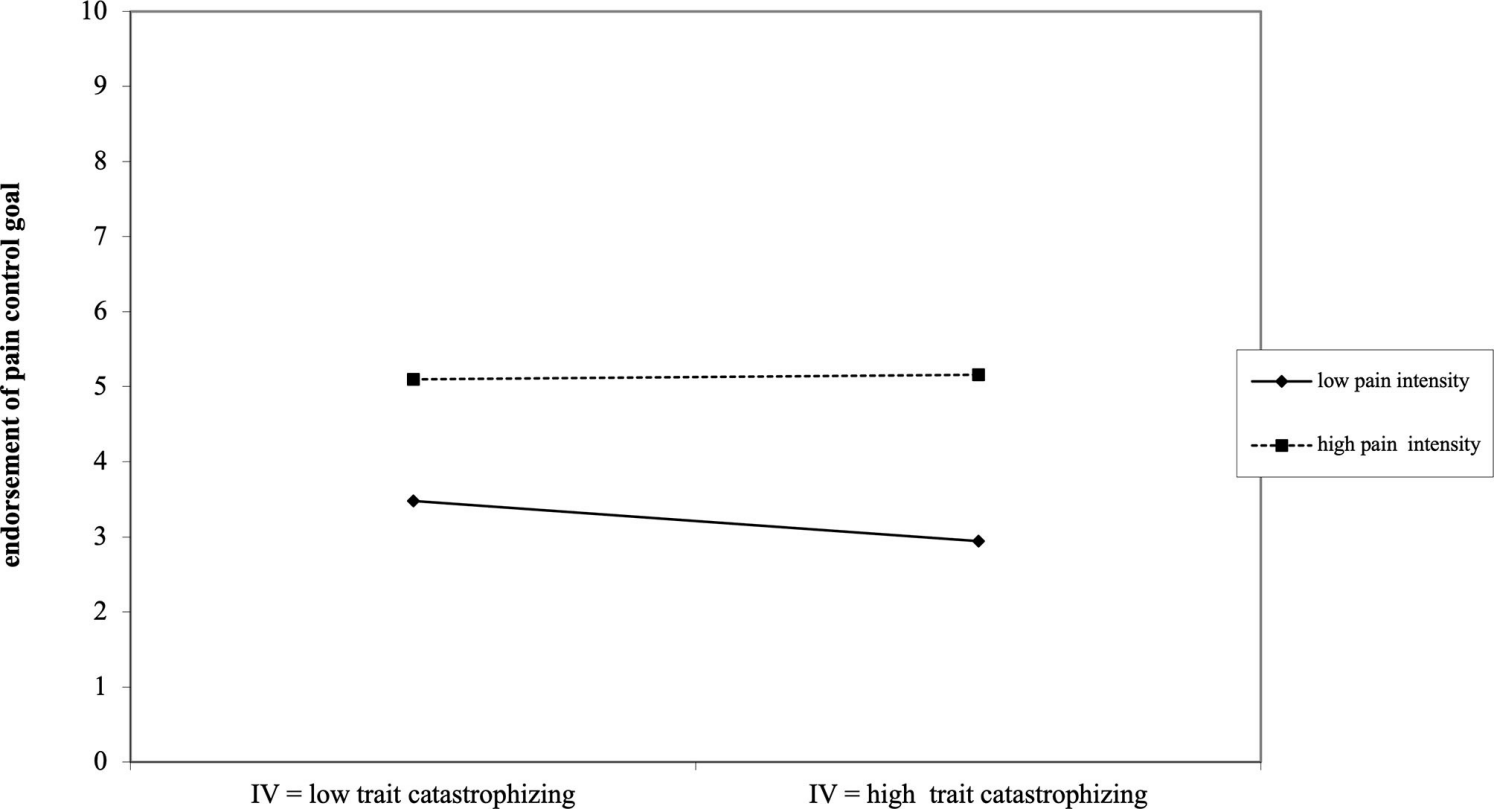
Depiction of how the significant interaction between general parental catastrophizing and perceived daily child pain intensity influences parental endorsement of the pain control goal.

Only the interaction between perceived daily child pain intensity and general levels of parental catastrophic thoughts (PCS-P general) was found to be significant [γ_110_ = 0.15; *t*_(350)_ = 2.39; *p* < 0.05, *r* = 0.18]. This significant positive interaction reveals no differences between parents with low and high levels of general catastrophizing on days that parents perceive high levels of pain intensity in their child, with all parents showing higher endorsement of the pain control goal on such days. However, on days where perceived child experiences of pain intensity is low, parents with high levels of general catastrophizing show a much more reduced focus on pain control compared to parents with low levels of general catastrophic thinking (see Figure 4). Results for the final hierarchical linear model assessing the impact of parental catastrophic thinking and perceived child pain intensity on parental endorsement of the pain control goal are presented in Table 3.

### Impact on Parents' Daily Endorsement of the Activity Engagement Goal

The intercept model indicated that 49.52% of the variance in parental priority for the activity engagement goal was accounted for by variables on the third level (between families or child characteristics), 46.44% by variables on the second level (parent characteristics), and 4.03% by variables on the first level (within parents or daily characteristics). The model exploring the main effects of the variables across all levels (steps 2–4) revealed a significant negative effect of child sex [γ_001_ = −2.56; *t*_(15)_ = −2.46; *p* < 0.05, *r* = 0.44], which indicates that parents endorse the activity engagement goal less for girls compared to boys. Furthermore, significant positive effect of daily parental catastrophic thinking (PCS-P daily) [γ_200_ = 0.29; *t*_(352)_ = 2.89; *p* < 0.01, *r* = 0.15] and perceived daily child pain intensity [γ_100_ = 0.41; *t*_(352)_ = 3.70; *p* < 0.001, *r* = 0.19] was found. No main effect of general parental catastrophic thinking (PCS-P general) was found. The random error terms for perceived daily child pain intensity and PCS-P daily were significant, so the interaction terms (perceived daily pain intensity^*^PCS-P daily and perceived daily pain intensity^*^PCS-P general) were added in step 5.

**Figure 5:**
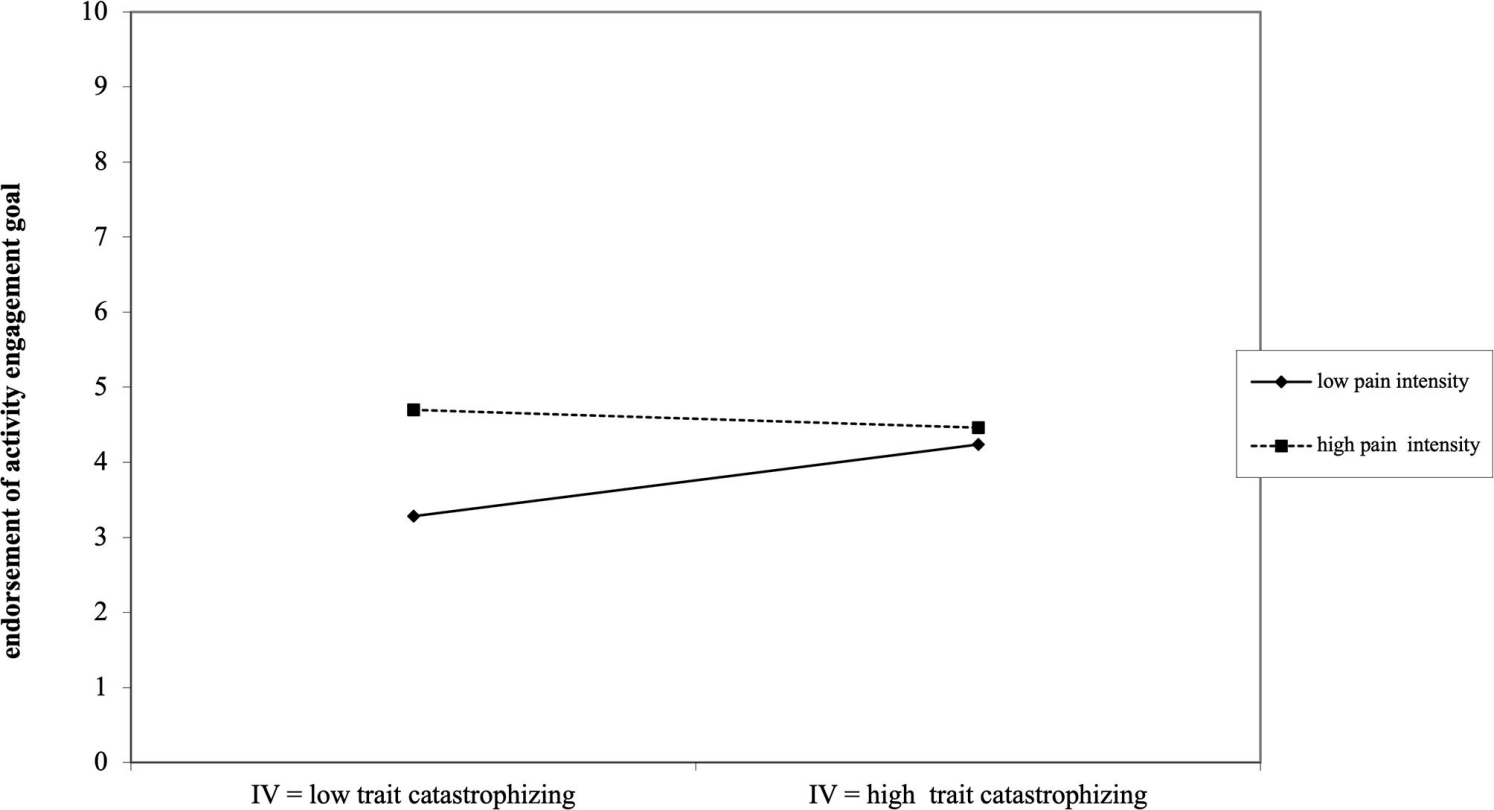
Depiction of how the significant interaction between general parental catastrophizing and perceived daily child pain intensity influences parental endorsement of the activity engagement goal.

Only the interaction between perceived daily child pain intensity and general levels of parental catastrophic thoughts (PCS-P general) was found to be significant [γ_110_ = −0.30; *t*_(350)_ = −3.59; *p* < 0.001, *r* = 0.18]. This significant negative interaction reveals that for parents with high general levels of catastrophic thoughts about their child's pain, the pursuit of the activity engagement goal is always moderately high and not influenced by perceived daily levels of child pain intensity. However, for parents with low general levels of catastrophic thinking, the focus on activity engagement shows more flexibility depending on the level of perceived child pain intensity: the lower the perceived child pain intensity, the lower the motivation to encourage activity engagement in their child (see Figure 5). Results for the final hierarchical linear model assessing the impact of parental catastrophic thinking and perceived child pain intensity on parental endorsement of the activity engagement goal are presented in Table 3.

## Discussion

Given the critical role parents play in understanding childhood chronic pain experiences, the current diary study explored how parental catastrophic thinking about their child's pain influences parents' daily experiences of distress and endorsement of pain control and/or activity engagement goals for their child, and how these associations were influenced by daily perceptions in fluctuations of child pain intensity. For parental levels of distress, our findings revealed that both the impact of general and daily parental catastrophic thoughts was moderated by perceived child's pain intensity levels. Daily fluctuations in perceived child pain intensity had the strongest impact for parents with low levels of general catastrophic thoughts about their child's pain (as measured by the PCS-P general), as increasing levels of perceived daily child pain intensity were related with increased levels of daily parental distress. However, for daily levels of parental catastrophic thoughts, the level of perceived child pain intensity mostly modulated parental distress on days where parents catastrophized a lot about their child's pain: on days where parents endorsed high levels of catastrophic thoughts, high levels of perceived child pain intensity were associated with higher levels of parental distress.

With respect to parents' daily goal pursuit, the findings illustrate how parents focus on both pain control and activity engagement on days when they have higher levels of catastrophic thoughts about their child pain. This was unrelated to the perceived levels of child pain intensity on that day. On the other hand, the impact of general catastrophic thoughts about their child's pain (i.e., PCS-P general) was influenced by the level of perceived pain intensity: on days where low levels of child pain intensity were perceived, a reduced focus on pain control was reported by parents with high levels of catastrophic thoughts (with no substantial change reported for the focus on activity engagement goals), while parents with low catastrophic thoughts rather reported a reduced focus on child activity engagement (with no substantial change reported for the pain control goal).

In sum, these results highlight how parental coping with their child chronic pain fluctuates considerably from day to day, with the impact of parental general tendencies (e.g., general levels of catastrophic thinking) moderated by daily perceived fluctuations in the child's pain experiences. Such daily differences underscore the need for continuous and situation specific assessment for a comprehensive understanding of how parents cope with their child's chronic pain. It is also interesting to note though that the variability in parents' daily coping responses was situated on different levels depending on the type of coping responses (i.e., emotional distress or goal endorsement). Indeed, for parental distress most variability was observed between days within the same parent, while for the endorsement of the pain control and activity engagement goal the variability was mostly observed between parents/families. This indicates that parental distress experiences are likely mostly influenced by situational characteristics of a particular day, while goal endorsement is mostly impacted by more stable characteristics of the parent and/or couple. While these preliminary observations need further confirmation, these findings could be of clinical relevance as it allows providing parents an insight on how their emotional responses are variable and changeable. Insight in such variability provides opportunities to teach parents appropriate emotion regulation techniques which would allow them to manage their daily changing levels of distress and engage in more optimal responses.

### Parental Daily Distress Experiences

For distress, the results are largely confirming accumulating evidence revealing how parents with high levels of catastrophic thoughts about their child experience heightened feelings of distress (e.g., Caes et al., 2012), as well as how daily levels of catastrophizing contribute stronger to parental emotional distress compared to general levels (Durand et al., 2017). However, our findings further untangle the complexity of these associations by demonstrating the strong moderating impact of daily fluctuations in parental perceptions of their child's pain experience, in particular pain intensity. Contrary to our hypotheses, this moderating impact was different for general vs. daily parental catastrophic thinking. As such our findings call into question the association between parental levels of general and daily catastrophic thinking, and possibly suggest that general and daily levels of catastrophic thinking influence parental responses independently.

The findings for daily levels of parental catastrophic thoughts are in line with our hypotheses, and previous literature, revealing how parental perceptions of high child pain intensity were associated with increased parental distress experiences on days where parents report high levels of catastrophic thinking. This finding further supports the assumptions of the model of empathy in the context of pain (Goubert et al., 2005) as well as the Interpersonal Fear Avoidance Model (Goubert and Simons, 2013) underscoring the interplay of child and parental characteristics in determining how parents emotionally cope with their child's chronic pain experiences. To our knowledge, however, this is the first study exploring these interpersonal interactions on a daily basis, thereby highlighting how the interplay of interpersonal characteristics may not necessarily be stable but fluctuate from day to day. It is likely to assume that these daily fluctuations may present a barrier and explain observed difficulties in skill generalization from the treatment sessions to the home context (Jensen et al., 2003; Guite et al., 2014). Clinically this can have important implications, as it not only emphasizes the need for situation-specific assessment to gather a comprehensive picture of parental coping responses but also the need to ensure any skills parents are taught to reduce their distress and catastrophic thoughts take into account such substantial situational influences in their responses. For instance, our findings highlight the need to encourage parents to practice emotion regulation and cognitive exercises aimed at challenging their catastrophic thoughts in various different situations in their home environment.

However, contrary to our expectations, parental perception of high child pain intensity contributed to more distress only in parents reporting low levels of general catastrophic thinking. While on face value this is unexpected, Figure 3 demonstrates how parents high on general thinking report high levels of distress independent of their perception of child pain intensity. Consequently, there is more variance and situational differences in the distress experiences of parents low in general catastrophic thinking. These results further corroborate the findings from a vignettes study in parents (Caes et al., 2012), which revealed that parents with high levels of general catastrophic thinking show less flexibility, compared to parents low in catastrophizing, in how they cope with their child's pain experience depending on the particular pain characteristics, e.g., short term vs. long-term pain or mild vs. intense pain. Therefore, it appears that parents with low levels of catastrophic thinking attune their feelings of distress appropriately to the particular pain experience of their child, while parents with high levels of general catastrophic thoughts rather show a sustained mild to high distress response, even in low threatening situations (i.e., low child pain intensity). Such sustained experiences of distress entail the risk of engaging in maladaptive responses toward their child pain (e.g., overprotectiveness, Caes et al., 2012) and developing clinical depression (e.g., decreased response to rewards and anhedonia) through elevated activity of the hypothalamus- pituitary-adrenal (HPA) axis (Willner, 2017). To avoid this chronic experience of distress, it is vital for parents with a high general tendencies to catastrophize about their child's pain to increase their distress tolerance by learning how to appropriately regulate their emotional coping responses in accordance with their child's specific pain characteristics (Guite et al., 2018). For instance, possible adaptive regulation strategy to mitigate the impact of parental distress and catastrophic thinking is mindfulness. Indeed, there is growing evidence on how mindfulness, or being focused on the present moment, in parents of a child with a chronic illness (e.g., diabetes) is an asset which allows parents to adjust their level of distress and protectiveness toward their child to the child's current needs (Van Gampelaere et al., 2019).

### Parental Daily Goal Endorsement

The findings with respect to parental goal endorsement are more complex and not completely in line with our hypotheses. For instance, the role of daily parental catastrophic thinking on their goal endorsements was not associated with the perceived level of the child's pain intensity on that particular day and revealed a counterintuitive simultaneous endorsement of both pain control and activity engagement goals on days parents report high levels of catastrophic thinking (as measures by the PCS-P daily). Based on previous evidence (Caes et al., 2012) and motivational theories on goal incompatibility, which highlight the need to prioritize one goal over the other (Riediger and Freund, 2004), it would be expected that the goals of pain control and activity engagement are deemed incompatible by parents and hence decisions on which goal will be prioritized would need to be made. Following such preliminary evidence (Caes et al., 2012) we expected that on days when parents reported high levels of catastrophic thinking (i.e., high levels of PCS-P daily), the endorsement of pain control goals would be stronger compared to the endorsement of activity engagement goals. It is possible, that this unexpected equal goal endorsement of potentially incompatible goals is related to the formulation of the items assessing parental goal endorsement. In particular, the item assessing activity engagement includes the subphrase “*even if he/she could experience pain by doing so”* which could have influenced parents' interpretation of the item in an unintended way. Indeed, this subphrase could have led parents to interpret this item as follows: “*I had to encourage my child to engage in his/her normal activities because he/she was in pain and hence did not want to engage in those activities by themselves”*. Given this potentially unintended interpretation, the positive association found between parents' activity engagement goal endorsement and daily parental catastrophic thinking could be a reflection of how parental catastrophic thoughts (in this case the daily levels) impact how much parents attune their coping responses to the child's pain characteristics. Following this reasoning, it is likely to assume that on days where parents report lower levels of catastrophic thoughts, they feel less threatened by the child's pain, and hence can accordingly attune their responses as reflected in a lesser need to encourage activity engagement. Alternatively, social desirability could also play a role in explaining this unexpected association. Future research is needed to entangle this complexity.

As for the impact of general or general levels of parental catastrophic thinking, a moderation by the fluctuations in perceived child pain intensity levels was observed with parents who report low levels of catastrophic thinking showing adjustments in the endorsement of the activity engagement goal. As explained above, the potentially unintended interpretation of the activity engagement goal endorsement item could explain the lower endorsement of the activity engagement goal on days the child's pain intensity was perceived as low, especially by parents with low general levels of catastrophic thinking. Indeed, the need to stimulate such engagement in daily activities could be reduced when the child is perceived to experience less pain as the child engages in those activities by themselves without the parents needed to encourage them. Particularly for parents with low levels of catastrophic thinking, who might be better at attuning their coping responses more to the child's pain characteristics, could a reduced perceived level of child pain intensity be related to lowering their endorsement of activity engagement that is relative to the child's pain experiences.

Although the above-described findings align with the proposition that parents with high levels of catastrophic thinking are less flexible in adjusting their goals (Caes et al., 2012), the reduced endorsement of the pain control goal by parents with high general of catastrophic thinking—compared to parents with low general levels—on days when the perceived child pain intensity is low challenges this proposition. It is not entirely clear how to interpret this finding and further confirmation of these findings in larger, more heterogenous samples, is needed to gain a comprehensive understanding of the factors that influence parental goal endorsements. The current study is the first to assess these associations on a daily basis in parents caring for a child with chronic pain, compared with the existing evidence stemming from a vignette methodology in parents of pain-free children, which could contribute to the differences. For instance, it is possible that the flexibility of parental coping responses might be dependent on the type of coping responses (i.e., emotional vs. goals and/or behavioral responses) and whether we focus on general vs. situation-specific or daily changing) parental characteristics. While speculative, and requiring testing, it is possible lower endorsement of the pain control goal in parents with high general catastrophic thinking on days with low perceived pain intensity is an attempt to regulate their continued heightened feelings of distress and compensate for their heightened focus on pain control goals on high intensity days. Such explanation would further strengthen the need for providing parents with high levels of catastrophic thinking the tools to appropriately regulate their feelings of distress.

A research avenue that can potentially shed light on the complexity of how perceived child pain intensity and parental catastrophic thinking influence parental goal endorsement, is to explore how these different and potentially incompatible parental goals translate into different parental behaviors. It is reasonable to assume that parental behavior toward their child pain is driven by multiple goals and, vice versa, a particular goal can be achieved through various different behaviors (Rasmussen et al., 2006). For instance, in order to reach their goal of reducing their child's pain parents could comfort their child or stimulate their child to distract themselves by engaging in different activities (e.g., watching a movie). Consequently, depending on the specific behavioral coping strategy the parent engages in, the goal of pain control and activity engagement are not necessarily incompatible on a behavioral level and can be achieved by the same behavioral response. There is growing evidence on how parents who catastrophize about their child's pain tend to engage in protective behavioral responses (e.g., reassuring, comforting and paying attention to their child's pain) at the expense of engaging the child in their daily activities (e.g., attending and engaging with school; Caes et al., 2011; Sieberg et al., 2011). Therefore, the goal of controlling their child's pain and engaging their child in activities might be incompatible for parents experiencing high levels of catastrophic thinking given that they do not naturally perceive coping-promoting responses, such as distraction, as a possible way to reduce their child's pain, leading. On the other hand, for parents low in catastrophic thinking achieving their pain control and activity engagement goal could be more compatible, given their tendency to engage more frequently in, and therefore perceive, coping-promoting behaviors as an approach to reduce their child's pain. However, as highlighted by the current study's findings, this incompatibility might also be influenced by daily fluctuations in parental catastrophic thoughts and perceptions of their child's pain intensity.

### Limitations

It is important to consider our findings in light of several limitations. The study was conducted in a relatively small and homogenous sample, limiting the generalizability of the findings in various ways. The relatively small sample prevented us from conducting intricate prospective analyses and rather limited our analytic approach to cross-sectional analyses. Furthermore, only for a handful of families we were able to collect data from both parents, making comparisons and explorations of potential differences between mothers and fathers impossible. Reflective of the typical population distribution of patients attending a pediatric chronic pain clinic, our sample comprised mostly of girls. Hence, recruiting through pediatric chronic pain clinics gives an over-representation of girls and to fully understand boy's experiences alternative recruitment routes might need to be explored. Additionally, the average age of the children in our sample was on the lower end (i.e., 9.7 years of age) of our target range (i.e., 8–14). This has important implications for our findings as younger children are more dependent on their parents, which could substantially influence parental goals as well as coping responses. Replication within an older sample of adolescents with chronic pain is advised. Lastly, given our focus on children with chronic headache or functional abdominal pain, the results may not be applicable to other pain conditions or different pain locations (e.g., musculoskeletal pain, sickle cell disease).

Beyond the limitations related to generalization of the findings, it is important to recognize that the level of daily child pain intensity was reported by the parent, rather than the child, thereby potentially introducing a reporting bias and preventing us from inferring causal effects. Previous evidence indeed highlights discrepancies between parent and child reports on the child's functioning, particularly when parents endorse high levels of catastrophic thinking (Birnie et al., 2020). Specifically, heightened levels of parental catastrophic thoughts have been found to be related to increased perception of child's pain intensity and disability (Birnie et al., 2016, 2020). While our findings align with a study utilizing child report (e.g., Neville et al., 2020), further research is needed to disentangle this complex interrelation between parental perceived pain intensity and catastrophic thinking.

## Conclusion

Despite these limitations, the findings underscore the importance of including parents into clinical pain management programs for pediatric chronic pain, with a focus on understanding the underlying mechanisms of and situational influences on parental engagement in maladaptive coping strategies. Accumulating evidence indeed shows promise of actively including parents within interdisciplinary pediatric pain management programs, by for instance addressing parental problem-solving skills (Palermo et al., 2016), parental distress tolerance and resilience (Russell et al., 2020), and parental psychological flexibility (Kemani et al., 2018). However, the observed daily fluctuations in parental emotional response and goal endorsements—and how these are associated with parental general and daily catastrophic thinking as well as child's daily pain intensity—reveals a strong need to ensure the acquisition of pain management skills within these dedicated pain management programs generalizes from the therapy to the home environment.

## Data Availability Statement

The datasets for this study are available upon requested by contacting the first and corresponding author (Line Caes).

## Ethics Statement

The study was approved by the Ethics Committees of the Faculty of Psychological and Educational Sciences of Ghent University, University Hospital Ghent, AZ Maria Middelares Ghent, and General Hospital Nikolaas, Belgium. Written informed consent to participate in this study was provided by the participants' legal guardian/next of kin.

## Author Contributions

LC designed the study procedures, assisted with data collection, took the lead on data analyses, and wrote up the results and discussion section. CG assisted with data collection and contributed substantially to the write up of the manuscript, taking the lead on writing up the methods section, and providing feedback on all other sections. EV, MV, and KK assisted with recruitment of the patients in their respective clinics and provided feedback on all sections of the manuscript. LG assisted with the design of the study procedures and contributed substantially to the write up of the manuscript, taking the lead on writing up the introduction section, and providing feedback on all other sections. All authors contributed to the article and approved the submitted version.

## Conflict of Interest

The authors declare that the research was conducted in the absence of any commercial or financial relationships that could be construed as a potential conflict of interest.
